# Implementation of eHealth and AI integrated diagnostics with multidisciplinary digitized data: are we ready from an international perspective?

**DOI:** 10.1007/s00330-020-06874-x

**Published:** 2020-05-06

**Authors:** Mark Bukowski, Robert Farkas, Oya Beyan, Lorna Moll, Horst Hahn, Fabian Kiessling, Thomas Schmitz-Rode

**Affiliations:** 1 Department of Science Management, Institute of Applied Medical Engineering, Helmholtz Institute, University Hospital Aachen, RWTH Aachen University, Pauwelsstr. 20, 52074 Aachen, Germany; 2 Fraunhofer Institute for Applied Information Technology, FIT, Schloss Birlinghoven, 53754 Sankt Augustin, Germany; 3 Chair of Computer Science 5 - Information Systems & Databases, RWTH Aachen University, Ahornstr. 55, 52074 Aachen, Germany; 4 RWTH Centralized Biomaterial Bank (RWTH cBMB), Institute of Pathology, University Hospital Aachen, RWTH Aachen University, Pauwelsstr. 30, 52074 Aachen, Germany; 5 Fraunhofer Institute for Digital Medicine, MEVIS, Am Fallturm 1, 28359 Bremen, Germany; 6 Institute for Experimental Molecular Imaging, Center for Biohybrid Medical Systems (CBMS), University Hospital Aachen, RWTH Aachen University, Forckenbeckstr. 55, 52074 Aachen, Germany; 7 Comprehensive Diagnostic Center Aachen, University Hospital Aachen, Pauwelsstr. 30, 52074 Aachen, Germany; 8 Institute of Applied Medical Engineering, Helmholtz Institute, University Hospital Aachen, RWTH Aachen University, Pauwelsstr. 20, 52074 Aachen, Germany

**Keywords:** Diagnosis, Information storage and retrieval, Artificial intelligence, Electronic health records, Europe

## Abstract

Digitization of medicine requires systematic handling of the increasing amount of health data to improve medical diagnosis. In this context, the integration of the versatile diagnostic information, e.g., from anamnesis, imaging, histopathology, and clinical chemistry, and its comprehensive analysis by artificial intelligence (AI)–based tools is expected to improve diagnostic precision and the therapeutic conduct. However, the complex medical environment poses a major obstacle to the translation of integrated diagnostics into clinical research and routine. There is a high need to address aspects like data privacy, data integration, interoperability standards, appropriate IT infrastructure, and education of staff. Besides this, a plethora of technical, political, and ethical challenges exists. This is complicated by the high diversity of approaches across Europe. Thus, we here provide insights into current international activities on the way to digital comprehensive diagnostics. This includes a technical view on challenges and solutions for comprehensive diagnostics in terms of data integration and analysis. Current data communications standards and common IT solutions that are in place in hospitals are reported. Furthermore, the international hospital digitalization scoring and the European funding situation were analyzed. In addition, the regional activities in radiomics and the related publication trends are discussed. Our findings show that prerequisites for comprehensive diagnostics have not yet been sufficiently established throughout Europe. The manifold activities are characterized by a heterogeneous digitization progress and they are driven by national efforts. This emphasizes the importance of clear governance, concerted investments, and cooperation at various levels in the health systems.

Key Points

• *Europe is characterized by heterogeneity in its digitization progress with predominantly national efforts. Infrastructural prerequisites for comprehensive diagnostics are not given and not sufficiently funded throughout Europe, which is particularly true for data integration.*

• *The clinical establishment of comprehensive diagnostics demands for a clear governance, significant investments, and cooperation at various levels in the healthcare systems.*

• *While comprehensive diagnostics is on its way, concerted efforts should be taken in Europe to get consensus concerning interoperability and standards, security, and privacy as well as ethical and legal concerns.*

**Electronic supplementary material:**

The online version of this article (10.1007/s00330-020-06874-x) contains supplementary material, which is available to authorized users.

## Introduction

The digitization of medicine with its increasing amount of heterogeneous data and technologies faces significant challenges and also offers a great potential for medical diagnostics [1–4]. In clinical routine, diagnostic information derives from various sources and is collected by medical doctors with different expertise. These comprise physicians who do the anamnesis and physical examination, radiologists, nuclear medicine specialists, pathologists, and experts in clinical chemistry and omics analysis. Their diagnostic findings are usually presented in board meetings (e.g., tumor boards), where diagnoses are rendered and therapeutic strategies discussed. Some disciplines already use computer-assisted (decision) support to facilitate data interpretation [5]. However, it can be expected that the integration of the entirety of diagnostic information from the different disciplines in one analysis tool that uses artificial intelligence (AI) will elucidate important new connections between the features and improve the diagnostic accuracy as well as the prognostic power of clinical examinations.

Unfortunately, in most European countries, the lack of a suitable information technology (IT) infrastructure in the hospitals and medical practices, the absence of high-quality curated data, and difficulties to access and exchange data are inhibiting the translation of integrated diagnostics into clinical routine and even research. Comprehensive diagnostic centers were founded to act as local seeding points at university hospitals to evaluate the value of integrated diagnostics for distinct disease entities [6]. However, for the broad implementation of eHealth and AI integrated diagnostics, these local centers do certainly not replace central organizations (at national or even international level) that ensure common structures, standards, and data safety.

The reasons for an unexploited potential of comprehensive approaches with a lack of clinical as well as research implementation are manifold, interconnected and concern infrastructural, technical, political, and ethical challenges. The complexity makes it difficult to keep up with the variety of approaches across Europe. Therefore, we provide insights into current international activities on the way to digital comprehensive diagnostics (CD). For this purpose, we analyzed European funding and international hospital scoring concerning digitization. Furthermore, the article includes a technical view on CD in terms of data integration and analysis. In this context, we discuss radiomics as an example of an evolving field with particularly high relevance for radiologists.

### Data integration and analysis

**Fig. 1:**
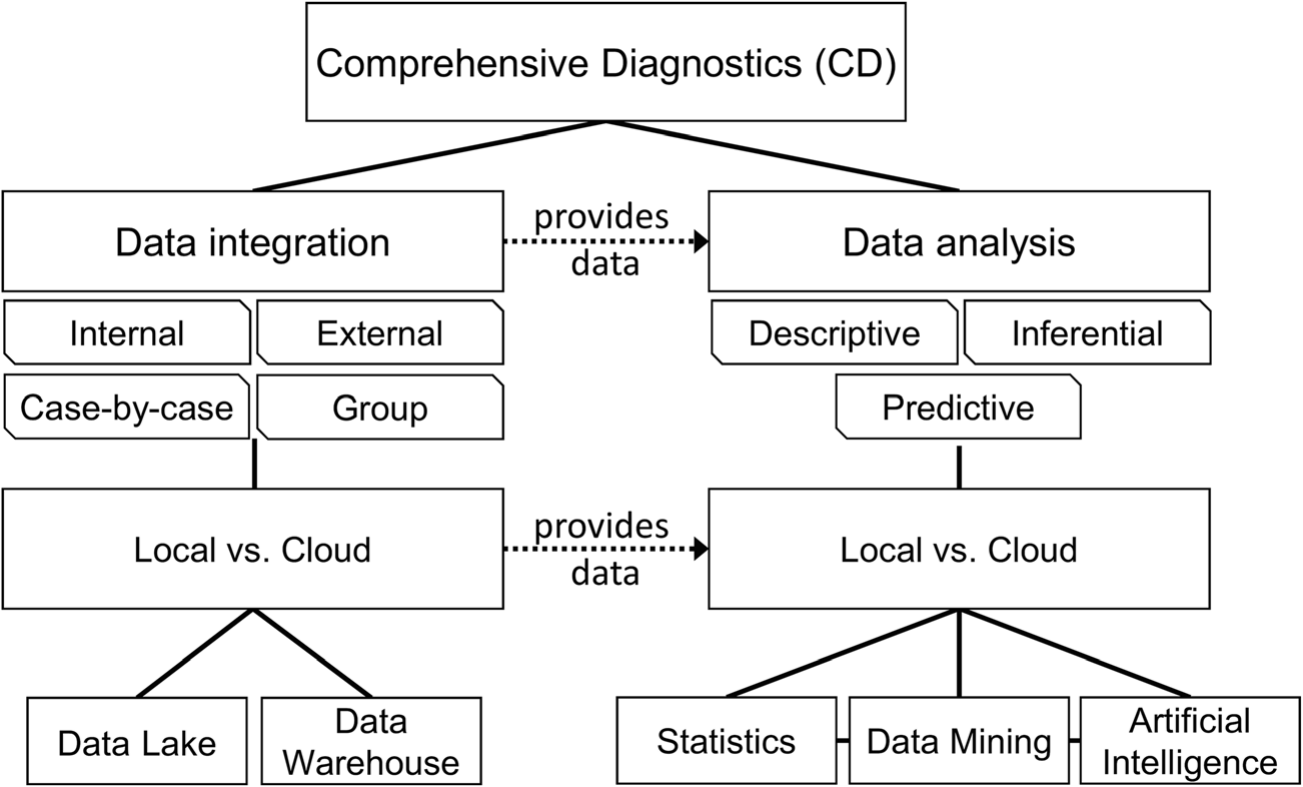
Overview of different data solutions for comprehensive diagnostics (CD) infrastructure. CD requires solutions for data integration and data analysis. Data integration can be performed based on own data (internal) or data imports (external) or a mixture of both. Data integration can be performed locally or in the cloud to build data warehouses or data lakes. One can also build the data bases from individual cases or groups. Data warehouses store organized data, which requires efforts of structuring and cleaning. Data lakes store raw data. Subsequent efforts need to be taken for the specific selection and organization of the data for each need/analysis. Data analysis can be performed on the integrated data. It can be descriptive (e.g., graphical presentation of data), inferential (concluding from the sample case to the collective), and predictive (pattern found in historical data are used to foresee the fate of present cases). These analyses can be performed locally or by cloud computing. For this purpose, statistical methods, artificial intelligence, and data mining are applied

On the way to digital CD, technical data infrastructures are fundamental and among others substantially connected to regulative aspects in terms of data ownership, data privacy, and ethics. Due to the high complexity of the field, in this work, we predominantly focus on the technical elements of CD (Fig. 1).

CD approaches are characterized primarily by the processing of heterogeneous health data. This demands for the integration of data from diverse sources as a prerequisite for their analysis. Currently, this is mostly done in the context of research. Its use in clinical routine would be much more restrictive, e.g., requiring the approval of the medical software and the related medical procedures. However, even research strongly depends on the availability of structured health data from the clinical routine [7]. Thus, much effort must be spent into IT infrastructures and their interoperability to facilitate comprehensive approaches and translational medicine with a “bi-lateral ‘two-way’ iteration between bench-side and bedside” [8].

#### Infrastructure and interoperability

Health data are generated and stored in highly diverse systems by heterogeneous stakeholders. However, due to the organization of most health providers, the established clinical procedures, and ethical and data ownership concerns, a huge amount of usable health data are currently trapped inside the organizational boundaries of private medical practices, hospitals, clinics, and within patients’ monitoring devices (e.g., smart watches). This disrupts the progress of comprehensive diagnostics. Many healthcare institutions implement centralized repositories by pooling data from multiple systems into data warehouses or data lakes [9, 10]. Sharing these data out of the organizations’ boundaries is not a viable solution since the anonymization of data may not be possible for certain data types, such as genomic data, and also since linking data sets increases the re-identification risk [11, 12]. Furthermore, research communities build domain-specific data infrastructures that cannot easily communicate with each other (e.g., biobanks) [13, 14]. The problem of accessing data outside the network remains, and since data are collected for a specific use and duplicated outside of the first data source, it limits the record linkage and integration of multimodal data. However, sharing health data offers great advantages, such as the improved comparability and reproducibility of the results of image data analysis between different sites [15]. International and national initiatives, such as European Open Science Cloud [16] or German Medical Informatics Initiative [17], are establishing research data infrastructures for supporting access to data and reuse of it. They provide federated environments of semantically interoperable and integrated data as well as related services for access and data analytics.

In this context, data stewardship in healthcare is gaining importance. It refers to the process of creation, (re)use, storage, and archiving of research and clinical data that aims to ensure data quality, integration, and reuse. This also includes reducing or eliminating data silos and protecting patient privacy [18]. The FAIR (findable, accessible, interoperable, and reusable) principles for scientific data management and stewardship provide guidance for data producers and infrastructures to improve discoverability and interoperability of data. A specific emphasis is on supporting the reuse by individuals and enhancing the ability of machines to automatically find and use the data [19, 20].

Data analytics is another challenge in this fragmented health data space. We would like to point out that the term “analytics” is used in various ways, often in the context of business intelligence, big data, and also predictive analysis. We use it as a synonym to “analysis” but want to distinguish analysis, processing, and use of data from their management and integration. To enable data-driven research, healthcare, and thus CD, there are approaches to support the distribution of analytics over distributed data (often related to the terms distributed analytics or federated learning). For this, new solutions such as grid/cloud computing have been proposed [21]. Moreover, software solutions such as i2b2 or DataShield support analyzing sensitive data in a distributed fashion [22, 23]. The Personal Health Train is another approach that improves the reuse of data by sharing analytics [24]. The core design principle is to give data owners the authority to decide and monitor the use of their data, e.g., in terms of access and purpose. Distributed data analytics utilizes the data at the original location, can interact with the data, and complete their task without giving access to the end-user. In contrast to other approaches, it is technology agnostic and aims at maximum interoperability between diverse systems, by focusing on machine-readable and interpretable data, metadata, workflows, and services [25] (Table 1).

**Table 1:** Comparing the data analytics solutions for integrated health data with a selection of exemplary activities. ETL stands for extract, transform, load

Features	Cloud computing	Data warehouses or lakes	Distributed analytics
Data Integration and Transformation	Data to be transferred and transformed into a central repository. ETL cost/effort comparably high.	Data to be transferred and transformed into a central repository. ETL cost/effort comparably high.	FAIR data points. ETL cost/effort comparably less.
Security	Data need to be exported to an external network and platform owner by different firms or entities. Rules and regulations should be checked.	Data reside in owner site but need to be moved into central server. Some data sources may be privacy sensitive and require encryption or anonymization before moving into central repository.	Data reside in actual source and owner has the authority. Authentication and authorization mechanisms need to be established between parties.
Speed	Network latency may occur.	No network latency issues.	Network latency may occur.
Scalability	Easy due to dynamic scaling model of cloud.	Need to purchase new software or hardware to accommodate large-scale growth.	Execution power requirement is distributed through different sites. Scalability requirement and extension cost may be less compared to central systems.
Data Integration	Assuming that all data are kept in a centralized manner, data integration will be easy.	Assuming that all data are kept in a centralized manner, data integration will be easy.	Data integration is done through executing aggregations on results coming from different nodes.
Cost	In general, cloud software is priced under a monthly or annual subscription, with additional recurring fees for support, training and updates. Considered as operating expenditure (an additional overhead cost the organization will continue to pay).	On-premise software is generally priced under a one-time perpetual license fee (usually based on the size of the organization or the number of concurrent users). There are recurring fees for support, training and updates. A capital expenditure (one large investment upfront).	Network cost
Reliability	Uptime and reliability are guaranteed through provider’s service agreement.	Dependent on the human resources and equipment and acquired support services.	Dependent on the reliability of each node in the systems. Multiple control centers reduce the risk of a system breakdown.
Exemplary activities	End of 2019 the National Health Service (NHS) Shared Business Services (SBS) in UK launched a cloud solutions framework valued at up to £500 m [26] that provides streamlined and OJEU (Official Journal of the European Union) compliant route to purchase cloud infrastructure and cloud optimization solutions for NHS and other authorities of the public sector. “The framework provides access to 24 carefully selected suppliers and offers bespoke and off-the-shelf solutions from cloud solution design and as-is assessment to end-to-end cloud solutions.” [27]	The Research IT at Stanford Medicine established a clinical data warehouse “STAnford Research Repository” (STARR) that integrates data of several clinics. 2018 STARR-Radio was introduced: a cloud scale radiology imaging repository that brings data from PACS into a research archive with different modalities, e.g., chest and breast X-ray, MRI, CT, and ultrasound. [28] 2019 STARR-OMOP was launched that includes EHR data from ~ 2.67 M patients. This data infrastructure is connected to Stanford Nero, which allows Big Data Analytics. [29]	Research partners from the Netherlands, Belgium, and Germany implemented an IT infrastructure “euroCAT” in five radiation clinics. They showed a proof-of-principle with the use-case of predicting severe dyspnea after radiotherapy for future “big data” infra-structures and distributed learning studies for personalized medicine. [30]

The market for software systems and individual solutions, in clinical and research environments related to data integration and analysis, is diverse (Table 2). It includes comprehensive infrastructural software solutions such as Electronic Medical Record (EMR) systems used by health providers, especially as part of hospital information systems. An EMR, which is also known as Electronic Health Record (EHR), includes digital patient records such as diagnostic and therapeutic data across time and medical fields. These EMR systems are the basis for generating integrated clinical workflow solutions and cross-disciplinary data exchange for research. Moreover, there are software solutions from IT giants such as Google for data analysis approaches [31], domain-specific popular software libraries such as OpenCV [32] in the field of computer vision, or tool and data repositories such as the Neuroimaging Informatics Tools and Resources Clearinghouse (NITRC) [33]. Further overviews can be found in reports such as “Magic Quadrant for Data Integration Tools” provided by Gartner [34].

**Table 2:**
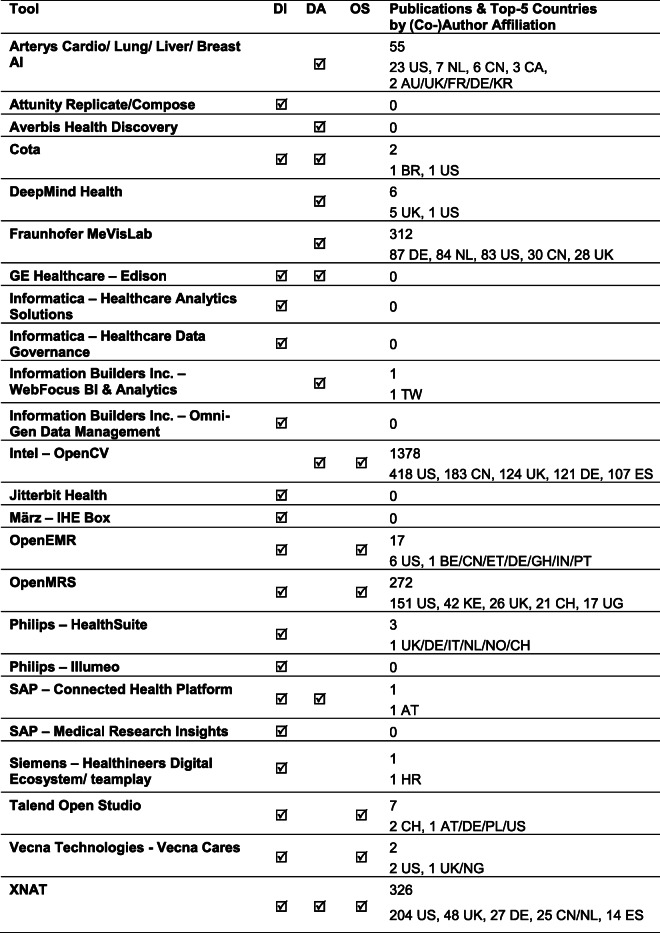
Selection of tools with capabilities in data integration (DI) and data analysis (DA). The tools are specified according to their open-source (OS) availability and their use in scientific publications together with the top 5 countries/regions according to their (co-)authorships. As an example: XNAT might be one suitable open-source tool for managing radiomics DICOM (Digital Imaging and Communications in Medicine) image data and clinical patient data supporting the HL7 (Health Level 7) FHIR protocol (Fast Healthcare Interoperability Resources) (see Supplement for further details on the methods)

Among the various health software systems and tools, in recent years, IBM Watson Health has been the subject of controversial debate [35, 36]. It is an AI-based software program designed for the decision support strongly focusing on oncology, clinical trials, and genomics with worldwide projects at 230 hospitals and health organizations [37]. Besides success stories such as recommending the best fitting breast cancer treatment [38] or enhancing next-generation sequencing [39], the projects also elucidated some problems for the clinical practice. For example, the MD Anderson Cancer Center first installed the EMR system from Epic System Corporation, which is besides Cerner Corporation one of the EMR market leaders in the USA [40]. However, the introduction of the Epic EMR system at MD Anderson was challenging in terms of time (~ 4 years), costs (up to 76.9% drop in adjusted income), and integration into clinical workflow [41–43]. The implementation of IBM Watson at MD Anderson failed with an estimated cost of $62 million due to mismanagement and problems regarding the integration with the EMR system [41, 44]. Projects in Germany and China with IBM Watson indicate obstacles to clinical applicability. This might be caused by the fact that the system was trained on data of the Memorial Sloan Kettering Cancer Center in New York [45–47]. These data may not directly translate to the other countries due to the different healthcare environment (e.g., regarding ethnical population, medical guidelines, and available treatments and medication). Those issues are related to the ongoing discussion about “poor data quality, incompatible datasets, inadequate expertise, and hype” holding up the big data revolution in healthcare [48].

In summary, we were not able to identify a ready-to-use solution, especially not for CD. The selection, implementation, and integration of software systems depend on the environment and its capabilities. With the increasing digitization, the growing amount of health data, and the upcoming new regulations, the tasks are becoming even more extensive, especially regarding comprehensive approaches. This makes standards and supporting regulations all the more important for the selection and use of IT systems and software tools. Furthermore, standards might unlock the so far restrained sharing of health data. A multitude of standards already exists for different domains (Table 3**)**. However, many of these are historically grown and co-exist in different versions (e.g., HL7v2.x, HL7v3, and HL7 FHIR) that might limit their interoperability. Furthermore, also the commercial implementation of standards like DICOM is heterogeneous, not fully compatible between different vendors and contains a different degree of information. This complicates their use for comprehensive approaches. Therefore, one needs to analyze the homogeneity and compatibility of each standard for CD, extinct redundancies, and try to reduce the overall number of standards despite the complicating large number of stakeholders involved [67].

**Table 3:** Selection of commonly used international standards and profiles for medical data documentation and communication. In addition, the use of the standards in scientific publications together with the top 5 countries/regions according to their (co-)authorships is shown (see Supplement for further details on the methods)

Acronym	Full name	Issued by	Purpose	Publications and top 5 countries by (co-)author affiliation
ATC [49]	Anatomical Therapeutic Chemical Classification System	WHO	Classification system for the active ingredients of drugs	5008: 811 US, 707 SE, 659 NL, 577 DK, 517 DE
DICOM [50]	Digital Imaging and Communication in Medicine	NEMA	Broadly accepted, open communication standard for encoding and exchange of medical image data and associated meta-information	12,149: 5012 US, 1245 CN, 913 DE, UK 860, 564 KR
EDIFACT [51]	Electronic Data Interchange for Administration, Commerce and Transport	UN/CEFACT (ISO 9735)	Open communication standard for the exchange of administrative data also healthcare IT systems	49: 8 UK, 7 DK, 5 FR/US, 3 NL/SE
HL7 V2 [52]	Health Level 7	HL7	Dominant communication standard for event notification between hospital IT systems	237: 89 US, 14 KR, 11 UK, 9 FR/DE
HL7 V3, CDA [53, 54]	Health Level 7, Clinical Document Architecture	HL7	Open documentation standard regarding the structure and content of clinical documents based on XML	626: 228 US, 38 DE, 32 KR, 20 UK, 18 SP
HL7 V4, FHIR [55]	Fast Healthcare Interoperability Resources	HL7	Fine granular communication standard for medical resources, data, and interfaces, including application programming interface (API) specification	450: 193 US, 33 DE, 28 UK, 25 CA, 14 NL
ICD [56]	International Classification of Diseases	WHO	Documentation standard for disease classification	52,948: 23,504 US, 6107 UK, 3271 CA, 2925 CN, 2800 TW
IHE PIX [57, 58]	Patient Identifier Cross Referencing	IHE	Integration profile for patient ID management based on HL7v2.x	30: 11 US, 6 DE, 4 CA, 2 CN, 1 BR/DK/SI/ KR/FR/PT/UK
IHE XDS [59]	Cross-Enterprise Document Sharing	IHE	Dominant interoperability profile for patient electronic health records	103: 28 US, 10 CA,7 AT, 6 DE/NL
ISO/IEEE 11073 [60]	Medical/Health Device Communication Standards	ISO/IEEE	Family of ISO, IEEE, and CEN joint standards addressing the interoperability of medical devices	45: 11 KR, 8 US, 7 SP, 5 UK, 3 IT
LOINC [61]	Logical Observation Identifiers Names and Codes	Regenstrief Institute	Documentation standard and ontology for laboratory data	1227: 630 US, 45 DE, 35 UK, 33 KR, 29 FR
MeSH [62]	Medical Subject Headings	NLM	Nomenclature enabling the indexing of medical publications	10,526: 3142 US, 1488 UK, 1304 CN, 1111 CA, 640 AU
RadLex [63]	Radiology Lexicon	RSNA	Comprehensive lexicon for standardized indexing and retrieval of radiology information resources	267: 166 US, 16 DE, 12 UK, 11 CN, 10 CA/FR
SNOMED CT [64]	Systemized Nomenclature of Medicine Clinical Terms	SNOMED Int.	Documentation standard for comprehensive, unified medical nomenclature comprising English and other languages	3717: 1529 US, 367 UK, 173 DE, 151 SW, 144 FR
UMLS [65]	Unified Medical Language System	NLM	Metathesaurus aiming at integrating all important medical terms	2829: 1408 US, 190 UK, 146 CN, 100 DE, 85 FR
XSPA [66]	Cross-Enterprise Security and Privacy Authorization	OASIS	eHealth profiles for Security Assertion Markup Language (SAML) and eXtensible Access Control Markup Language (XACML)	15: 4 DE/US, 3 SP, 1 AT/BE/CA/PL/TW

#### Radiomics

Radiomics is a part of CD and describes the extraction of quantifiable features from radiologic images and its analysis by machine or deep learning [68, 69]. The combination of genomic and radiomics features is described by the term radiogenomics [70]. However, the term radiogenomics is also applied to describe the use of genomic data to refine radiotherapy. Thus, there are ambiguities in the terminology and some authors even suggest dividing it further into radiogenomics, radioproteomics, radiolipidomics, etc. Nonetheless, everything targets towards integrated diagnostics supported by AI. The evolving field of radiomics (Table 4) presents multiple publications reporting every year about the diagnostic power of radiomics analyses [75–80].

**Table 4:** Overview of radiomics generations from handcrafted features to end-to-end learning and delta radiomics

Radiomics generation	Technical details	References
1st	Few, well-understood handcrafted features	Kumar et al 2012 [71]
2nd	Large number of generic, deterministic features	Aerts et al 2014 [69]
3rd	Self-learning deep CNNs as feature extractor	Khalvati et al 2018 [72]
4th	End-to-end deep learning integrates both feature extraction and classification/prediction	Hosny et al 2018 [73]
Delta	Based on temporal feature changes instead of single time points, can be combined with all radiomics generations	Fave et al 2017 [74]

However, there is an increasing discussion about the reliability of publications due to the data quality in terms of heterogeneity of datasets and parameters, sample size, and risk of bias regarding patient selection [81, 82]. This concern could be partially addressed by centralized databases continuously integrating huge amounts of data from various sites. Although in some countries like Denmark, Sweden, Finland, Austria, UK, Switzerland, and Spain, the nationwide implementation of eHealth is strongly promoted by the government, a common conduct has not yet been defined in Europe, which is highly demanded.

**Fig. 2:**
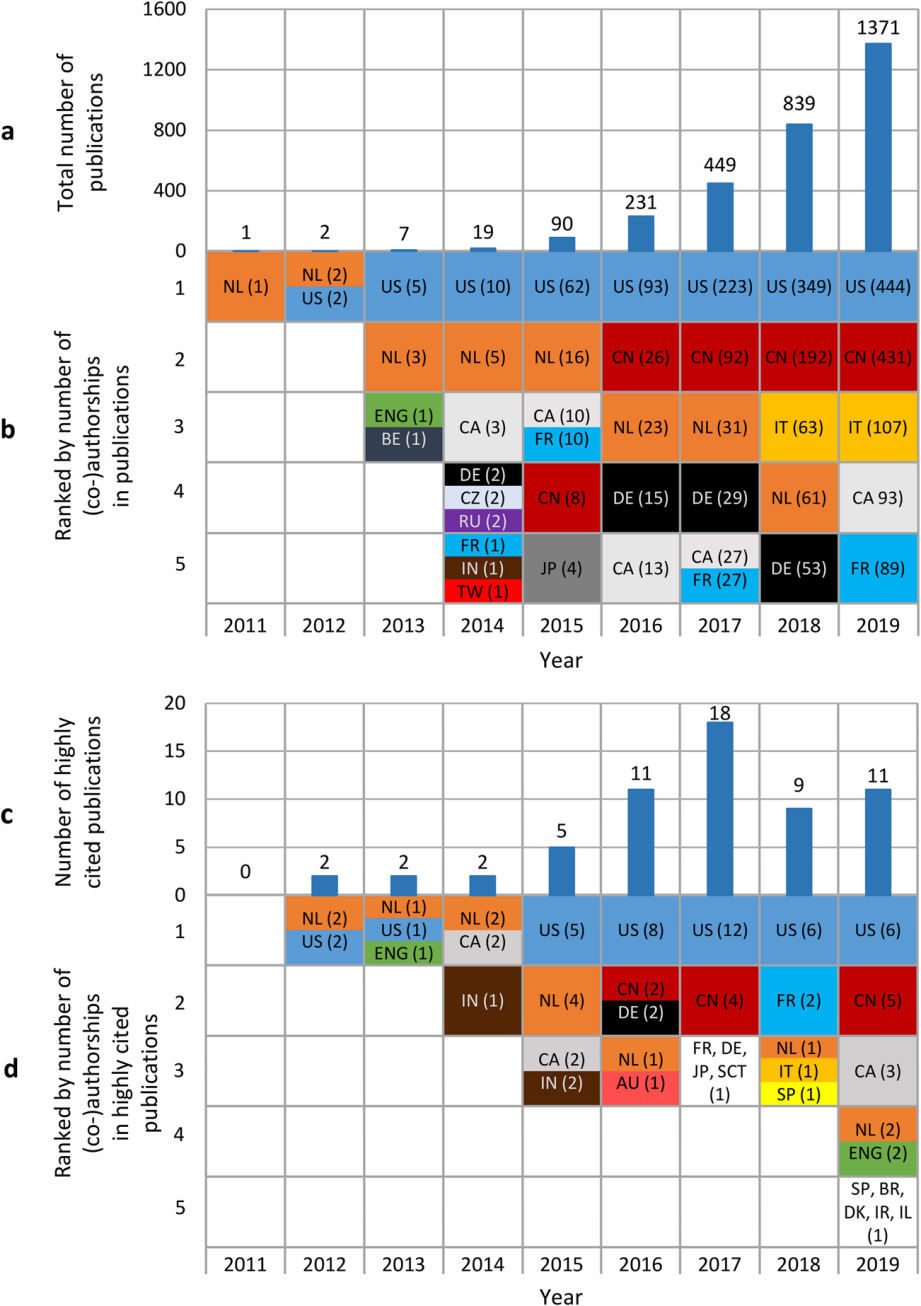
Annual international publication activity in radiomics from 2011 to 2019 (total 3009) based on a Web of Science search. **a** Number of publications. **b** Top 5 countries ranked by their number of (co-)authorships in publications (e.g., in 2012 there were two publications, both NL and US were involved, so each of them has two co-authorships in the two publications of 2012). **c** Number of highly cited publications (top 1% of the citations). **d** Top 5 countries ranked by their number of highly cited (co-)authorship publications (in total 60 high cited publications with 167 citations on average) (the methods and table of highly cited publications are part of the Supplement)

Our analysis of publications shows that the international research activity in radiomics is substantially increasing (Fig. 2). Between 2011 and 2019, 3009 radiomics publications have been indexed in the Web of Science, of which more than 73% have been published within the last 2 years. Article frequencies put Italy (the Netherlands before 2018) in the leading position in Europe and in third place in the worldwide ranking behind the USA and China.

**Fig. 3:**
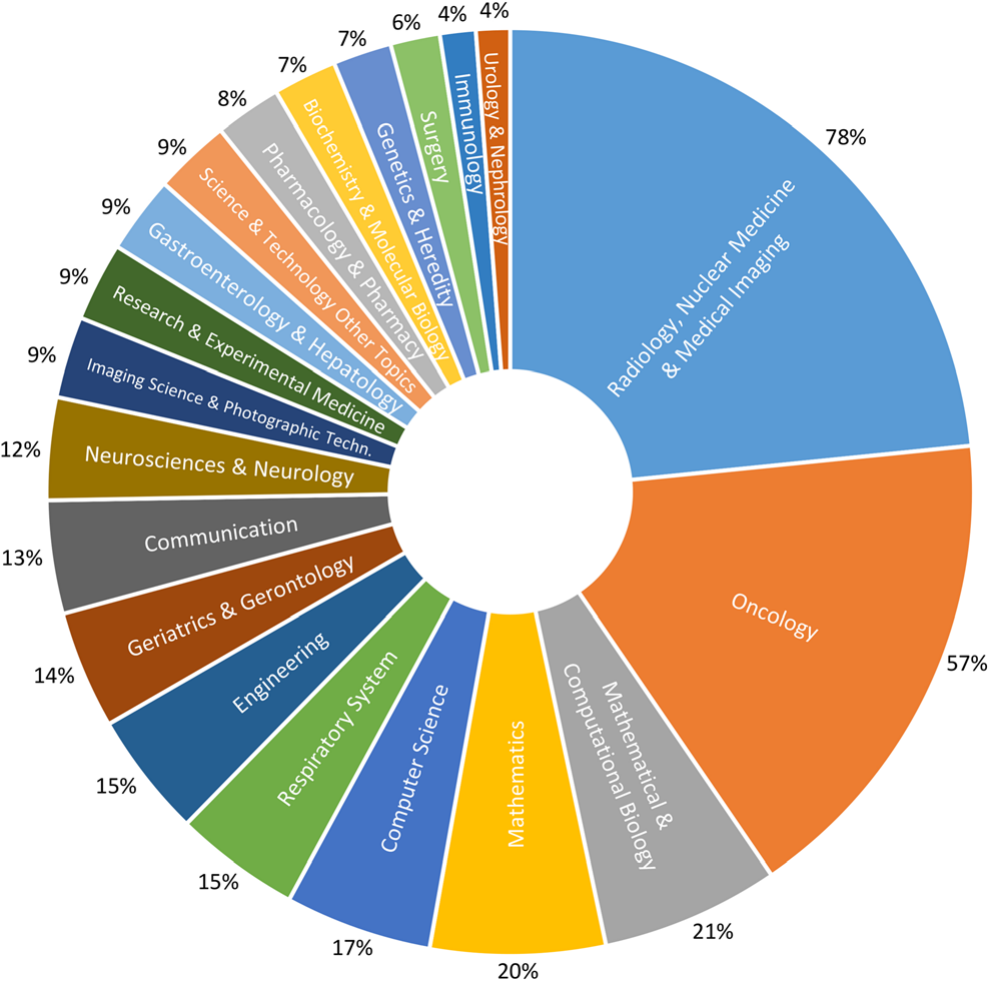
Share of 3009 radiomics publications in the clinical and technical research areas assigned by the Web of Science for 2011 to 2019. Multiple assignments of research areas per publications are possible (see Supplement for further details on the methods)

With the increase in publications related to radiomics since 2015, the publication activity is characterized by multiple research fields with clinical and technical topics (Fig. 3). A screening of the publications showed the dominance of the clinical topic “oncology” and the lack of additional data sources besides medical images in the sense of comprehensive diagnostics.

In this context, there is a wide range of tools for data viewing and analysis, both in-house solutions and online available software that is often based on MATLAB (e.g., MITK, 3D-Slicer, IBEX, itk-SNAP, TexRAD, CERR). Since other fields like digital pathology, genomics, and proteomics are evolving and also show their potential for CD, foundations need to be laid for consolidating the different data qualities in the ongoing digitization processes.

### Digitization of the healthcare systems

The basis for the implementation of CD including data integration and analysis in healthcare and research lies in the digitization. For an insight, we evaluated EU funding as well as the digitization of hospitals.

The EU funded 330 health-related projects in the Horizon 2020 [83] and FP7 [84] framework with a total budget of approximately €1.67 billion since 2015 regarding data integration, data analysis, and radiomics (Table 5). Considering the number and budget of projects, UK, Spain, the Netherlands, Germany, and Italy are at the forefront in data integration and analysis. There are more projects with industry coordination in data analysis; however, the relative budget shares are larger for data integration. Radiomics has played a minor role in EU funding so far. However, in agreement with the publication activity, the Netherlands has the biggest EU funding fraction in radiomics. Besides UK, other nations (e.g. Italy, France, and Germany) are hardly represented.

**Table 5:** Analysis of European funding in health-related topics for data integration, data analysis, and radiomics. In this context, budget, industrial participation, and geographical hotspots based on Horizon 2020 and FP7 projects (not finished before 2015) were considered. *Industrial participation includes public private partnerships (PPP) such as the German Research Center for Artificial Intelligence (DFKI) (see Supplement for further details on the methods)

	Data integration (DI)	Data analysis (DA)	Radiomics	Total
By number of projects				
Total number of projects	26	304	5	330
Top 10 geographical hotspots	DE (7) ES (5) UK (4) IT (2) NL (2) CH (2) LU (1) PL (1) FI (1) IL (1)	UK (45) DE (35) NL (34) ES (33) IT (20) FR (17) EL (15) CH (13) DK (12) IL (12)	NL (4) UK (1)	UK DE ES NL IT FR CH EL IL DK
% of projects coordinated by industry*	23%	32%	40%	31%
By project budget (budget rounded in million €)				
Total budget of projects	164	1006	13	1170
Top 10 geographical hotspots	ES (72) DE (29) UK (26) IT (14) NL (8) IL (7) CH (5) FI (2) LU (0.4) PL (0.1)	NL (167) UK (140) ES (127) DE (106) EL (71) IT (67) CH (63) FR (49) DK (43) FI (37)	NL (9) UK (4)	ES NL UK DE IT EL CH FR DK FI
% of budget of projects coordinated by industry*	29%	18%	20%	19%
Top 3 industrial partners* (DI and DA budget > €9 million; radiomics budget > €2 million)	GlaxoSmithKline Alacris Theranostics Sintea Plustek	F. Hoffmann-La Roche Philips German Research Center for Artificial Intelligence (DFKI)	ptTheragnostic	F. Hoffmann-La Roche GlaxoSmithKline Philips

**Fig. 4:**
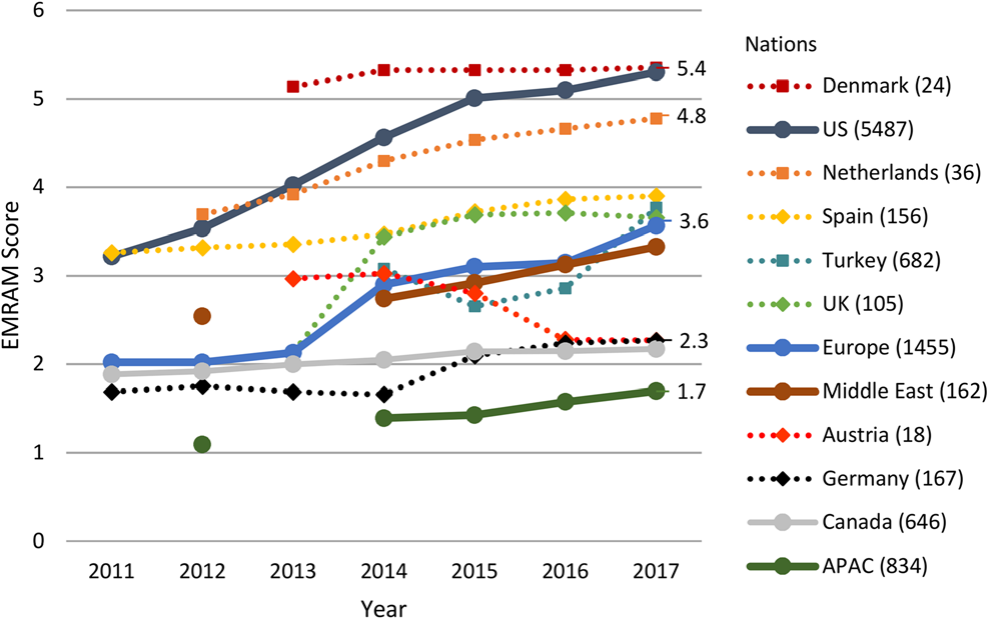
Degree of digitization of different countries’ hospitals based on the annually averaged EMRAM Score provided by HIMSS Analytics. Since in 2018, the criteria of the EMRAM stages were slightly modified and recent data are not yet available, we present data evaluated between 2011 and 2017. The eight-stage EMRAM Score ranges from 0 “paper-based” to 7 “paperless with data analytics” and it considers specific aspects such as closed-loop medication management. Besides single European nations, also United States (US), Middle East, Canada, and Asia-Pacific (APAC) are included. The numbers on the right represent the EMRAM Scores from 2017. In addition to the countries, the numbers of hospitals with EMRAM Score in 2017 are indicated. We would like to point out that due to the different number of hospitals assessed with the EMRAM Score, only a tendency can be evaluated (see Supplement for further details on the methods)

In relation to all projects as a whole, our financing comparison shows that the focus of the funded project activities is clearly on data analysis, while data integration ranks far behind. Based on this, one may conclude that the prerequisites for data integration are already in place. Therefore, we analyzed the maturity of EMR systems in hospitals in order to gain an impression of their degree of digitization. The basis for this is the evaluation with the EMR Adoption Model (EMRAM) provided by HIMSS Analytics [85] (Fig. 4), which allows the analysis across countries and regions over several years. There are multiple other maturity models in the health sector especially for the “management of information systems and technologies” [86]. However, these models are predominantly limited to partial aspects of digitization in the hospital, they are focused on local analysis, or they lack data over time and across multiple countries/regions.

**Fig. 5:**
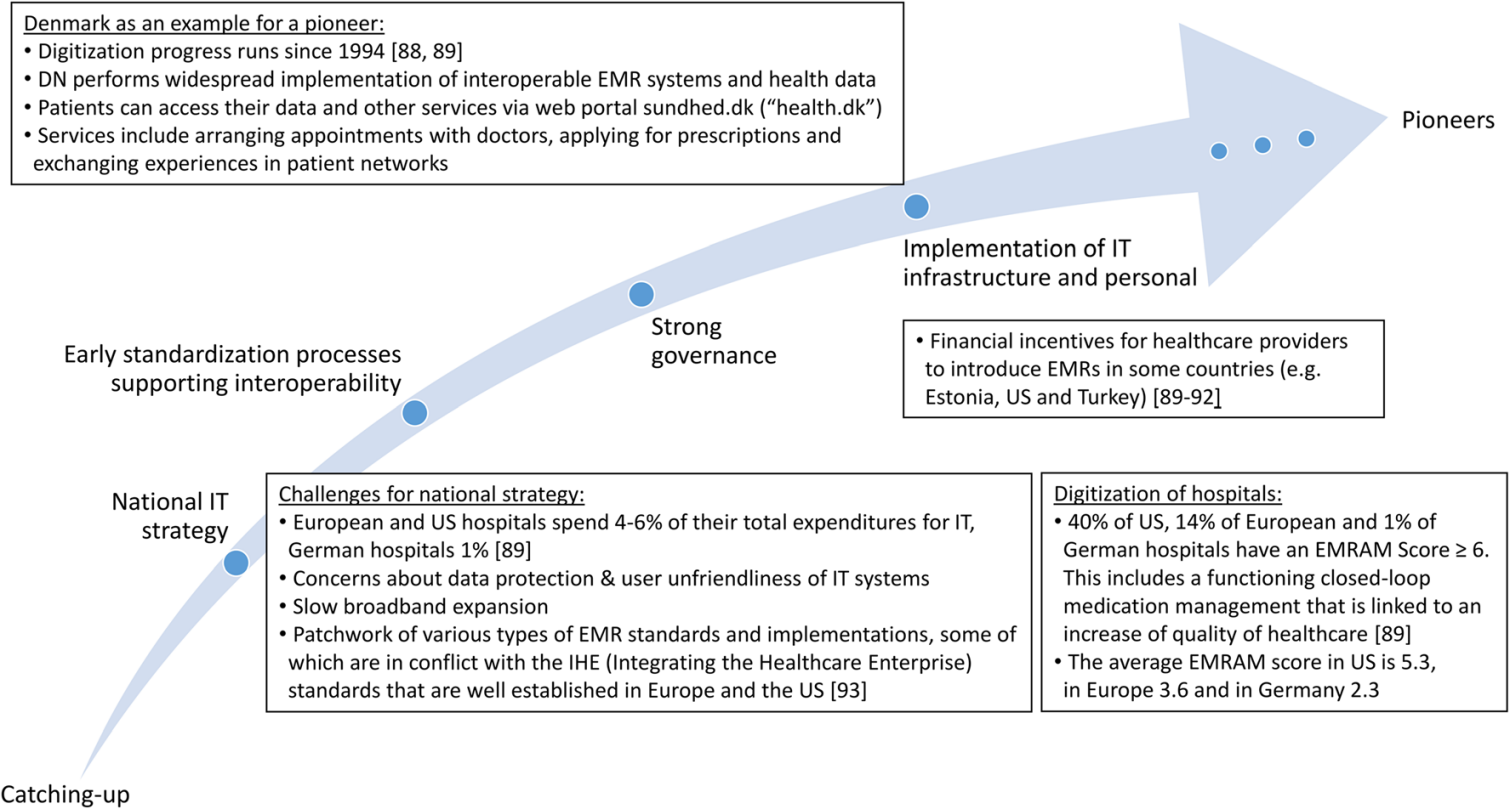
Challenges and implemented solutions for the digitalization of national healthcare systems including examples of countries at different stages of evolution

The comparison shows a heterogeneity in the digitization of European hospitals. There are pioneers such as the Nordic countries (e.g., Denmark and Estonia) and the Netherlands, which keep up with the US level. For example, Denmark already has a countrywide functioning IT infrastructure, which may explain that they are not represented in EU funding projects related to data integration. However, they are among the top 10 in funded data analysis projects. The Netherlands and Spain with increasing scores above the European average are strongly represented in EU funding and are still evolving their infrastructure. Furthermore, there are countries such as Germany that are lagging behind and try to catch up with a greater EU funding share and national efforts but “with varying degrees of enthusiasm and success” [87]: e.g., the German Medical Informatics Initiative comprises a national funding with more than €150 million for the development of data integration centers since 2018 [17], UK announced a £37.5 million investment in digital innovation hubs, and Japan released a law “to increase shared use of EMR data” [87]. Figure 5 illustrates the measures and challenges towards a digital healthcare system.

Our findings show the heterogeneity of (catching-up) activities related to data integration, so that the basis for comprehensive approaches has not yet been created throughout Europe. This is also in line with a recent study about integrated care programs in Europe [94] and with the Annual European eHealth Survey 2018 [95] that identifies diverse eHealth priorities: e.g., Germany has the highest priority in EMR implementation and UK in improving clinical access to information. The survey states that the top 3 challenges of healthcare providers are funding, standards for interoperability, and IT security.

The EU shows awareness to “support digital transformation of health and care in the EU by seeking to unlock the flow of health data across borders” [96] with a recommendation on an EMR exchange format [96]. This also relates to the potential risks for patients and public in terms of privacy, consent, and further aspects such as representative data and algorithmic bias [97]. A recent data leak of medical images and data from more than 5 million patients emphasizes the importance of data security and privacy [98]. Furthermore, the European Commission provides general ethics guidelines for trustworthy artificial intelligence [99], but without a legally binding consensus in Europe. These aspects also concern the current practice in medicine, which relies on guidelines that are evidence-based, practice-oriented recommendations.

Guidelines refer to groups and subgroups of diseases but they do not represent the level of the individual patient. Furthermore, guidelines are “conservative.” They reflect the current state of knowledge with a time delay, required to provide evidence and consensus. In contrast, AI has the potential to act more “progressive” and faster, with a finer granularity, because a diagnostic or therapeutic concept could be determined based on the entire data basis and all available frame conditions of an individual patient. However, its evidence level is vague. This raises some questions: How do physicians act in the area of conflict between “guideline truth” and AI-based recommendation? Could AI contribute to the process of generating and updating guidelines? Could this establish a new quality of evidence?

These questions are also linked to the new challenging topic of explainability of AI results as they derive from a “black box” [67, 100, 101]. In terms of the European General Data Protection Regulation (GDPR), the legal existence and feasibility of “a ‘right to explanation’ of all decisions made by automated or artificially intelligent algorithmic systems” are in doubt [102]. It becomes even more complex, when continuously learning and modifying AI systems in the clinical environment might no longer correspond to the initially approved system [103]. In this regard, the first attempts to approve such medical products and to standardize the process have been initiated by the US Food and Drug Administration (FDA) [104, 105]. They might serve as role models for Europe [103].

## Conclusion

Comprehensive approaches in diagnostics and their clinical implementation are still in their early stages because the prerequisites for digital medicine have not yet been sufficiently created throughout the European health systems. The manifold international activities are characterized by the heterogeneity of the European progress in digitization and driven by national efforts. Therefore, it is difficult to predict when and how most questions will be answered. Besides the leading examples, there is currently still a patchwork of systems and regulations as well as isolated solutions, which is why the effort for individuals in both clinical and research environments remains high. This emphasizes the importance of clear governance, investment, and cooperation at various levels in the healthcare system for the catching-up nations and institutions. These activities are crucial to overcome the multiple hurdles such as digital infrastructure, interoperability, security, and privacy as well as ethical and legal concerns for the benefit of research, healthcare, and ultimately patient health.

## Electronic supplementary material

ESM 1 (DOCX 47 kb).

## Abbreviations

AI: Artificial intelligence
APAC: Asia-Pacific
API: Application programming interface
ATC: Anatomical Therapeutic Chemical Classification System
CD: Comprehensive diagnostics
CDA: Clinical Document Architecture
CEN: European Committee for Standardization (Comité Européen de Normalisation)
DBLP: Digital Bibliography & Library Project
DFKI: German Research Center for Artificial Intelligence
DICOM: Digital Imaging and Communications in Medicine
EDIFACT: Electronic Data Interchange for Administration, Commerce and Transport
EHR: Electronic health record
EMR: Electronic medical record
EMRAM: Electronic medical record adoption model
ETL: Extract, transform, load
FAIR: Findable, accessible, interoperable, and reusable
FDA: US Food and Drug Administration
FHIR: Fast Healthcare Interoperability Resources
FP: Framework Programmes for Research and Technological Development
GDPR: EU General Data Protection Regulation
HL7: Health Level 7
IEEE: Institute of Electrical and Electronics Engineers
IHE: Integrating the Healthcare Enterprise
ISO: International Organization for Standardization
IT: Information technology
LOINC: Logical Observation Identifiers Names and Codes
MeSH: Medical Subject Headings
ML: Machine learning
NEMA: National Electrical Manufacturers Association
NHS: National Health Service
NITRC: Neuroimaging Informatics Tools and Resources Clearinghouse
NLM: National Library of Medicine
OASIS: Organization for the Advancement of Structured Information Standards
OJEU: Official Journal of the European Union
PIX: Patient Identifier Cross Referencing
PPP: Public Private Partnerships
RadLex: Radiology Lexicon
RSNA: Radiological Society of North America
SAML: Security Assertion Markup Language
SBS: Shared Business Services
SNOMED CT: Systemized Nomenclature of Medicine Clinical Terms
STARR: STAnford Research Repository
UMLS: Unified Medical Language System
UN/CEFACT: United Nations Centre for Trade Facilitation and Electronic Business
WHO: World Health Organization
WoS: Web of Science
XACML: eXtensible Access Control Markup Language
XDS: Cross-Enterprise Document Sharing
XML: Extensible Markup Language
XSPA: Cross-Enterprise Security and Privacy Authorization
