# Supplementary material for: Implementation of eHealth and AI integrated diagnostics with multidisciplinary digitized data: are we ready from an international perspective?
Source: Eur Radiol. 2020 May 6;30(10):5510–24. doi: 10.1007/s00330-020-06874-x (PMC7476980; doi:10.1007/s00330-020-06874-x)
Supplement: Supplementary file 1 — (DOCX 47 kb). [file 330_2020_6874_MOESM1_ESM.docx]

**Supplement**

**Materials and methods**

The analysis of the current activities in comprehensive diagnostics involves on the one hand the synthesis of existing research and reports based on literature and web search via Web of Science (WoS) [1], NCBI PubMed [2], and Google [3]. On the other hand, it comprises data analysis on radiomics publications, European funding, and hospital digitization. In the following further information about the materials and methods used in our work are explained along the article’s sections.

**Infrastructure and Interoperability**

The literature research includes the literature review on the secondary use of health data for research. Institutional solutions, research data infrastructures, distributed data analytics solutions as well as data stewardship and governance were reviewed.

Furthermore, to identify companies and tools, a web search for ‘big data integration’, ‘data integration’, ‘AI’, ‘deep learning’, and, ‘data mining’ in the healthcare sector by using ‘healthcare’ or ‘medical’ was performed. Afterwards, detailed information about the tools such as open source or proprietary, data analysis, and data integration were obtained from the website of the companies that offer the tools. Characterization of the tools by data integration and/or data analysis was performed according to the information given on each website. For literature research PubMed, NCBI PubMed Central (PMC) [4] as well as Digital Bibliography & Library Project (DBLP) [5] were used. In this context, compared to PubMed, PubMed PMC provided a higher amount of publications due to the full text search. The literature search using DBLP was performed in order to identify the amount of publications that can be found in a widely used computer science bibliography. The literature search was conducted using the tool name, sometimes in combination with the company name in case the tool name was too general such as Health Discovery. In all cases, the peak values were found in PubMed PMC. In addition to the number of publications, the top-5 countries/regions according to their
(co-)authorships are based on the WoS Results Analysis Tool [6]. The same procedure was used for identifying the use of standards in publications.

**Radiomics**

For the analysis of the current publication activity of different countries in the field of radiomics we searched in March 2020 at WoS with the search query ‘radiomics OR radiomic’ within all databases in the topic field, and extracted the annual country related results including the provided indicator of ‘highly cited papers’ (top one percent in each of the 22 Essential Science Indicators) and the assigned research areas with the WoS Results Analysis Tool [6]. The size of the patient cohorts and the quality of the publications are not considered, since this would exceed the scope of this article. Furthermore, we present absolute numbers and derived ranks, which are not adjusted to the size of the countries and the number of habitants.

**Digitization of the healthcare systems**

**EU funding**

We analyzed the European R&D activities related to the field of comprehensive diagnostics by focusing on EU projects concerning technical elements in terms of data integration and data analysis (e.g. artificial intelligence, decision support, radiomics) in a medical environment.

For this purpose, we used the EU project data of Horizon 2020 and FP7, which are provided by CORDIS. The datasets are publicly available on the European Union Open Data Portal [7,8]. To analyze recent R&D activities, only projects whose duration is at least partly in the period from 01.01.2015 were included. The term-based search was performed in 2019 July: first, a health-related domain selection was done using Apache Solr [9] as search and indexing tool with the following query ‘*health* OR *medic* OR *clinic* OR *therap* OR *diagnos*’ (13.290 records). Secondly, for the content-related analysis of radiomics, data integration, and data analysis the result set was queried within Solr based on the following keywords: ‘radiomics’, ‘big data’, ‘machine learning’, ‘artificial intelligence’, ‘decision support’, and ‘data integration’ (in total 516). Finally, a manual screening and validation of the result set lead to 330 records. This process also included the identification of projects coordinated by industry including public private partnerships. The results were evaluated with Microsoft Excel.

**Hospital digitization**

For the analysis of the degree of international hospitals’ digitization, we analyzed the data provided by HIMSS Analytics [10]. It includes the most recent and complete hospital’s eight-stage EMR Adoption Model (EMRAM) Score distribution for the period from 2011 to 2017 covering Europe, US, Middle East, Canada, Asia-Pacific (APAC) as well as Austria, Denmark, Germany, Netherlands, Spain, Turkey, and United Kingdom. HIMSS Analytics provided us the EMRAM Scores for each country/region for a specific year with the number of surveyed hospitals for the country/region, with the distribution to the eight-stage model and with the annual average. Therefore, we used the provided values and validated them by calculating the mean scores per year and per country/region.

Further European comparisons in terms of digitization of the health system, e.g. on policy activity, technical implementation, readiness, and actual usage of health data, can be found in a German study conducted by the Bertelsmann Stiftung in 2018 [11].

**Further information**

Table 6: List of highly cited publications of radiomics (2011-2019) based on the Web of Science search performed in March 2020.

| **DOI** | Year of publication |
| --- | --- |
| 10.1007/s00330-018-5787-2 | 2019 |
| 10.1158/0008-5472.CAN-18-2791 | 2019 |
| 10.1136/gutjnl-2018-316204 | 2019 |
| 10.1183/13993003.00986-2018 | 2019 |
| 10.1109/JBHI.2018.2825027 | 2019 |
| 10.1093/annonc/mdz001 | 2019 |
| 10.3322/caac.21552 | 2019 |
| 10.1016/j.jtho.2018.11.023 | 2019 |
| 10.1007/s11547-018-0951-y | 2019 |
| 10.1158/1078-0432.CCR-18-1305 | 2019 |
| 10.1016/j.radonc.2018.10.027 | 2019 |
| 10.1016/j.ijrobp.2018.05.053 | 2018 |
| 10.1016/S1470-2045(18)30413-3 | 2018 |
| 10.1158/0008-5472.CAN-18-0125 | 2018 |
| 10.1038/s41568-018-0016-5 | 2018 |
| 10.1148/radiol.2018172361 | 2018 |
| 10.1016/j.jacr.2017.12.026 | 2018 |
| 10.1016/j.jacr.2017.12.028 | 2018 |
| 10.3174/ajnr.A5391 | 2018 |
| 10.1016/j.lungcan.2017.10.015 | 2018 |
| 10.1038/nrclinonc.2017.141 | 2017 |
| 10.1158/0008-5472.CAN-17-0339 | 2017 |
| 10.1007/s00330-016-4637-3 | 2017 |
| 10.1038/sdata.2017.117 | 2017 |
| 10.1038/s41598-017-10649-8 | 2017 |
| 10.1148/rg.2017170056 | 2017 |
| 10.1158/1078-0432.CCR-16-2910 | 2017 |
| 10.1158/0008-5472.CAN-17-0122 | 2017 |
| 10.3348/kjr.2017.18.4.570 | 2017 |
| 10.1016/j.ejmp.2017.05.071 | 2017 |
| 10.1093/annonc/mdx034 | 2017 |
| 10.1093/neuonc/now256 | 2017 |
| 10.1186/s13058-017-0846-1 | 2017 |
| 10.1038/s41598-017-00665-z | 2017 |
| 10.1002/mp.12123 | 2017 |
| 10.1016/j.ejrad.2016.09.005 | 2017 |

| 10.1259/bjr.20160665 | 2017 |
| --- | --- |
| 10.1016/j.crad.2016.09.013 | 2017 |
| 10.1148/radiol.2016152234 | 2016 |
| 10.1158/1078-0432.CCR-16-0702 | 2016 |
| 10.1001/jamaoncol.2016.2631 | 2016 |
| 10.1148/radiol.2016152110 | 2016 |
| 10.1148/radiol.2016160845 | 2016 |
| 10.1088/0031-9155/61/13/R150 | 2016 |
| 10.1200/JCO.2015.65.9128 | 2016 |
| 10.1016/j.radonc.2016.04.004 | 2016 |
| 10.3389/fonc.2016.00071 | 2016 |
| 10.1038/srep23428 | 2016 |
| 10.1148/radiol.2015151169 | 2016 |
| 10.1097/RLI.0000000000000180 | 2015 |
| 10.1038/srep13087 | 2015 |
| 10.1038/srep11075 | 2015 |
| 10.1038/srep11044 | 2015 |
| 10.1016/j.radonc.2015.02.015 | 2015 |
| 10.1371/journal.pone.0102107 | 2014 |
| 10.1038/ncomms5006 | 2014 |
| 10.3109/0284186X.2013.812798 | 2013 |
| 10.1007/s00259-012-2247-0 | 2013 |
| 10.1016/j.mri.2012.06.010 | 2012 |
| 10.1016/j.ejca.2011.11.036 | 2012 |

**References**

1. Clarivate Analytics (2019) Web of Science (WoS); Available via https://www.webofknowledge.com. Accessed 08 Jul 2019.

2. National Library of Medicine (2019) PubMed; Available via https://www.ncbi.nlm.nih.gov/pubmed. Accessed 08 Jul 2019.

3. Google (2019); Available via https://www.google.com. Accessed 08 Jul 2019.

4. National Library of Medicine (2019) PubMed Central (PMC); Available via https://www.ncbi.nlm.nih.gov/pmc. Accessed 08 Jul 2019.

5. University of Trier and Schloss Dagstuhl - Leibniz Center for Informatics (2019) Digital Bibliography & Library Project (DBLP); Available via https://dblp.uni-trier.de. Accessed 08 Jul 2019.

6. Clarivate Analytics (2019) Web of Science Core Collection: Data Mining using 'Analyze Results'; Available via https://clarivate.libguides.com/woscc/analyze. Accessed 18 Jun 2019

7. EU Publications Office - EU Open Data Portal (2019) CORDIS - EU research projects under FP7 (2007-2013); Available via http://data.europa.eu/euodp/de/data/dataset/cordisfp7projects. Accessed 08 Jul 2019.

8. EU Publications Office - EU Open Data Portal (2019) CORDIS - EU research projects under Horizon 2020 (2014-2020); Available via http://data.europa.eu/euodp/de/data/dataset/cordisH2020projects. Accessed 08 Jul 2019.

9. Apache Software Foundation (2019) Apache Solr; Available via https://lucene.apache.org/solr/. Accessed 08 Jul 2019

10. HIMSS Analytics (2019) Electronic Medical Record Adoption Model; Available via https://www.himssanalytics.org/EMRAM. Accessed 27 Jun 2019.

11. R, Deimel L, Schmidtmann D, Piesche K, Hüsing T, Rennoch J et al. (2018) #SmartHealthSystems: Digitalisierungsstrategien im internationalen Vergleich; Available via https://www.bertelsmann-stiftung.de/de/publikationen/publikation/did/smarthealthsystems/. Accessed 26 Aug 2019
